# Time to Look for Ergonomically Viable Designs of Radiation Protection Aprons and Thyroid Shields in Orthopedic Surgery: A Survey of 416 Orthopedic Surgeons

**DOI:** 10.7759/cureus.48426

**Published:** 2023-11-07

**Authors:** Ashish Jaiman, Jatin Prakash, Rajesh K Chopra, Devdatta S Neogi

**Affiliations:** 1 Central Institute of Orthopedics, Vardhman Mahavir Medical College and Safdarjung Hospital, New Delhi, IND; 2 Department of Orthopedics and Trauma, Glan Clwyd Hospital, Betsi Cadwaladr University Health Board, Rhyl, GBR

**Keywords:** orthopedic surgeon, thyroid shield, radiation protection apron, lead apron, back pain

## Abstract

Introduction

The advent of minimally invasive surgery has increased the use of C-arm among orthopedic surgeons. Their views on the ergonomicity of radiation protection aprons and thyroid shields need elucidation. To investigate, we deliberated a question-based survey. The primary aim of the survey was to find out the percentage of those not using these devices, the prevalence of back pain, and its relationship with the type of radiation protection aprons.

Materials and methods

This was a cross-sectional survey. A five-section Google Forms survey (Google, Inc., Mountain View, CA) was filled out, and responses from 416 orthopedic surgeons were included. Analysis was carried out using Statistical Package for the Social Sciences (SPSS) version 14.0 (SPSS Inc., Chicago, IL).

Results

Of the total number of orthopedic surgeons, 36.8% felt that apart from radiation exposure, wearing a radiation protection apron was the biggest problem in C-arm usage. Furthermore, 20.4% wore thyroid shields the majority of the time. The 31-40 years age group was the most comfortable wearing these devices, wore them more often, and suffered more often from back pain (all p<0.01).

Conclusion

The study concluded that the majority of orthopedic surgeons were not comfortable with the current designs of radiation protection aprons and thyroid shields. Thyroid shields are worn less than aprons. Lead apron weight and thyroid shield ergonomicity were the number one reason for being bare-bodied. Among those who regularly wore aprons, a large proportion suffered from back pain.

## Introduction

In the last few decades, there have been leaps of improvement in surgical techniques and implants in the field of orthopedic surgery. Surgeons’ constant drive for perfection led to numerous innovations. C-arm is one such technological advancement and is no longer a luxury but a basic necessity for bone and joint surgery. However, the radiation exposure involved in its usage is the cost that surgeons have to pay [1].

The current radiation protection equipment, the aprons and the thyroid shields, have been designed to shield surgeons from secondary radiation [1,2]. Conventionally, these aprons were lead-based, but due to their weight, lead-free aprons have also been introduced [3,4]. Despite different designs, there have been numerous reports of back pain and increased fatigability among interventionists using these aprons, and a lack of education is evident regarding their use [5]. However, no large survey has been reported from orthopedic surgeons, who are conventionally treated as a low-risk group, as they use these aprons only for a limited time [2]. The dawn of minimally invasive surgeries has amplified C-arm usage among orthopedic surgeons, and the literature is deficient in views of the orthopedic community on the ergonomicity of the design of radiation protection aprons and shields, ease of usage, back pain prevalence, and its relationship with the usage of radiation protection apron. To investigate the above deficiencies in the literature, we planned to do a question-based survey. The primary aim of the survey was to find out the number of surgeons not using radiation protection equipment, the reasons for not using it, the prevalence of back pain among orthopedic surgeons who use lead aprons, and its relationship with radiation protection aprons and shields. This survey would be able to clarify the reasons why surgeons avoid using aprons and thyroid shields and would help companies modify designs according to surgeons’ comfort. Furthermore, the survey has also identified the characteristics of the “at-risk” population that avoid wearing these protective equipment, on whom hospital administration/radiation safety officers can focus and impart necessary knowledge to increase the use of radiation protection aprons and shields.

## Materials and methods

This was a cross-sectional survey study. At our institute, surveys that do not involve patients do not need ethical committee approval. A five-section Google Forms survey (https://docs.google.com/spreadsheets/d/16rjB8dkBOSbSXj53hjJQckI8h7S-e4VAiEgwDLRAWQU/edit) (Google, Inc., Mountain View, CA) was sent using e-mail and WhatsApp to over 700 orthopedic surgeons whose contact details were obtained from the national orthopedic registry. A waiting period of 11 days was allowed for all respondents to reply, following which the survey was closed. All surgeons who were actively practicing orthopedics and gave their consent to use their views for research purposes were included in the study. All those surgeons who did not give consent or submitted forms that were not complete were excluded from the study. A total of 441 responses were then studied.

The survey had five sections. The questions were finalized after active discussion among stakeholders. The first section asked if the respondent was an orthopedic surgeon. In the case of a non-orthopedic surgeon, the survey was closed. The second section collected basic information about the surgeon, such as age, gender, country of origin, and involvement in C-arm surgeries. The third section aimed at quantifying C-arm usage and the number of hours for which the surgeon wears the radiation protection apron and shield. The fourth section was based on questions that would analyze the problems that the surgeons face while wearing these devices. The final section dealt with the problem of back pain and the number of days missed due to back pain. Available replies were tabulated in an Excel sheet (Microsoft Corp., Redmond, WA), which was then statistically analyzed.

Statistical analysis

Statistical analysis was performed using Statistical Package for the Social Sciences (SPSS) version 14.0 (SPSS Inc., Chicago, IL). All quantitative data were expressed as mean ± standard deviation. The chi-square test, Fisher’s exact test, and one-way analysis of variance (ANOVA) were used to correlate between study variables such as the presence of back pain and the area of the practice with age, number of surgeries, and days missed due to back/neck pain. We also looked into factors for comfortable design. A p-value of less than 0.05 was considered statistically significant. All aspects of the statistical analysis were reviewed by a statistician. The goal of our analysis was to identify statistically significant differences between surgeons who wore radiation protection equipment, found it comfortable, and did not experience back pain and those who did not wear it, found it uncomfortable, and did experience back pain. Age, practice region, the number of C-arm surgeries each week, the number of hours per week spent wearing an apron, and the use of thyroid shields were used as criteria for grouping the data. These groups were compared based on characteristics such as how comfortable an apron and thyroid shield are to wear, whether surgeons wear aprons and thyroid shields, whether they experience back and neck pain, and how many days they missed work owing to these conditions.

To determine the most comfortable and the most faulty designs, we further evaluated the data using the chi-square test. The subgroup that never used this equipment received extra attention, and statistical tests of significance were employed to identify the traits unique to this group.

## Results

**Figure 1:**
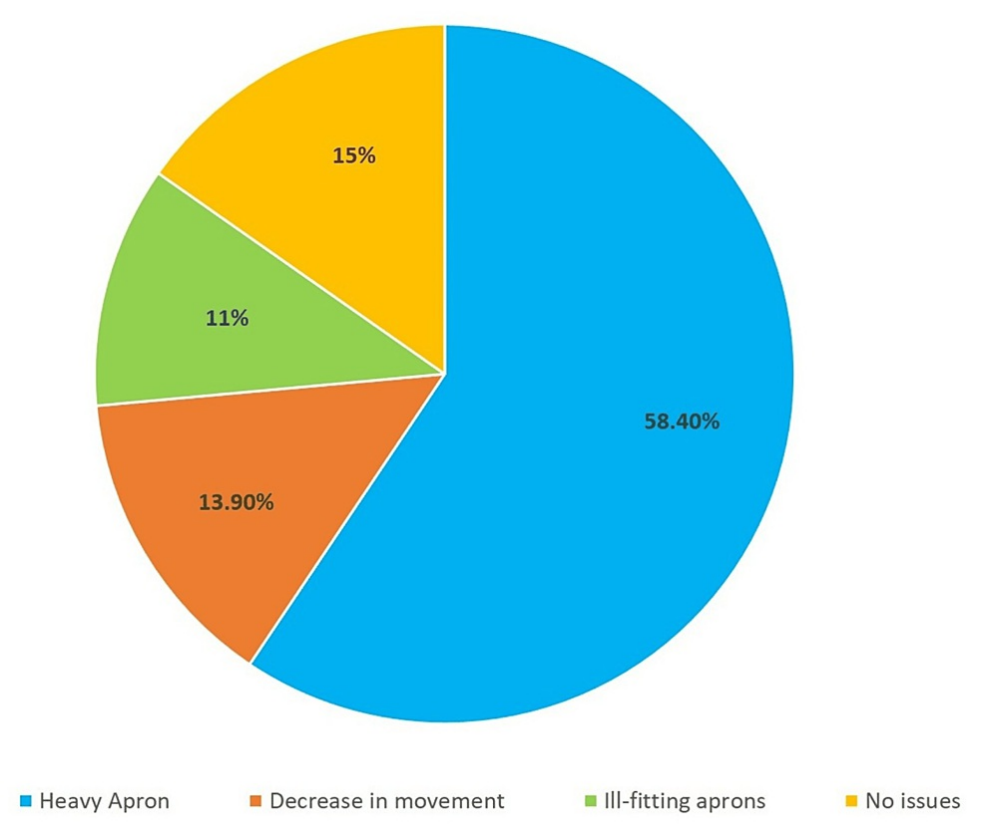
Problems while using C-arm (apart from radiation exposure)

A total of 441 responses were received for our Google Forms survey, of which 416 (95.2%) were from orthopedic surgeons. Of the orthopedic surgeons, 100% used C-arm in their practice. Of these, 153 (36.8%) felt that apart from radiation exposure, wearing a lead apron was the biggest problem in C-arm use. A total of 379 (91.1%) surgeons wore aprons the majority of the time. The biggest problem reported by surgeons for apron use was the heaviness of lead aprons reported by 243 (58.4%) surgeons, followed by a decrease in movements during surgery reported by 58 (13.9%) surgeons. Other problems reported by surgeons were that aprons were not fitting (11%). Only 40 (9.6%) surgeons reported that they were satisfied with the current design (Figure 1).

In contrast, only 20.4% (85) wore thyroid shields the majority of the time. Of the respondents, 142 (34.1%) found thyroid shields suffocating, and 114 (27.4%) found them to irritate the skin. Furthermore, 22% felt that it did not fit properly, and about 1% felt it affected their communication. Only 32 (7.7%) had no problems with the current design.

Forty-two (10.1%) of our respondents often forgot to wear aprons, and 155 (37.3%) always forgot to wear thyroid shields.

Another important problem reported with current aprons was that they make surgeons sweaty during surgery, with 326 (78.4%) surgeons complaining about this in our survey. Furthermore, it was interesting to note that surgeons in the government sector complained of sweatiness more often than in the private or mixed sector (p=0.000) (Table 1).

**Table 1:** Cross tabulation between areas of practice and feeling of sweatiness

			Does wearing a radiation protection apron make you sweaty during surgery?					
			No response marked	Half the time	Less than half the time	Majority of the time	Never	Total
What is your area of practice?	None mentioned	Count	1	0	0	0	0	1
		% within What is your area of practice?	100%	0%	0%	0%	0%	100%
	Government	Count	1	15	5	111	1	133
		% within What is your area of practice?	0.8%	11.3%	3.8%	83.5%	0.8%	100%
	Mixed	Count	0	9	2	35	0	46
		% within What is your area of practice?	0%	19.6%	4.3%	76.1%	0%	100%
	Private	Count	1	30	20	180	5	236
		% within What is your area of practice?	0.4%	12.7%	8.5%	76.3%	2.1%	100%
	Total	Count	3	54	27	326	6	416
		% within What is your area of practice?	0.7%	13%	6.5%	78.4%	1.4%	100%

**Figure 2:**
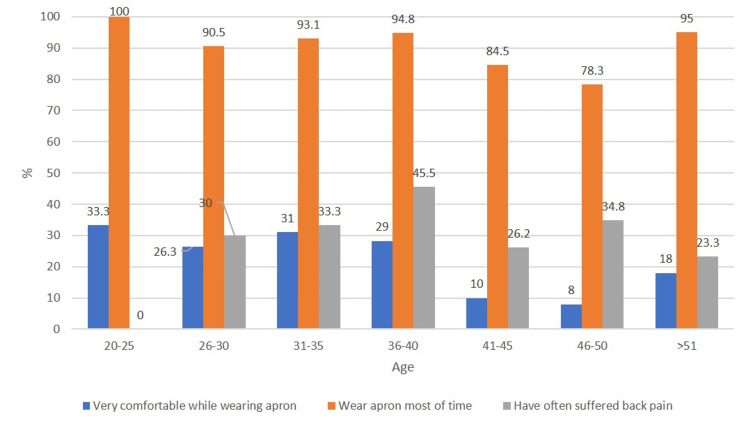
Bar diagram showing the relation of age to comfortability of wearing aprons, whether surgeons wore aprons, and the incidence of back pain

We found that the 31-40 years age group was most comfortable in wearing radiation protection equipment (p<0.01), wore it more often than the other age groups (p<0.01), and suffered more often from back pain (p<0.01) (Figure 2).

The extremes of age, despite having more usage, suffered from less back pain as there were only three responses in the 20-25 years age group, which is not representative of the general population, so this age group was not considered, and although >51 years wore lead apron more often than other groups, they suffered from less back pain as the number of surgeries performed by this age group was less (41.3% performed less than three surgeries per week).

**Figure 3:**
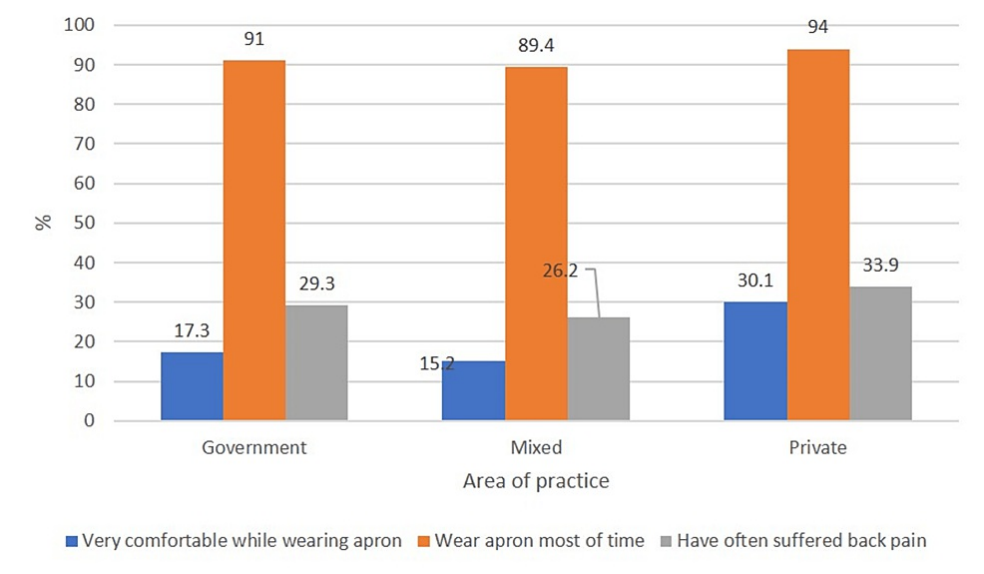
Bar diagram showing the relation of the area of practice to comfortability of wearing aprons, whether surgeons wore aprons, and the incidence of back pain

The area of practice also influenced the above factors. Private practice surgeons were most comfortable with radiation protection equipment (p<0.01), donned it more frequently (p<0.01), and experienced more back pain (p<0.01) than government practice surgeons (Figure 3).

Furthermore, surgeons performing 7-10 surgeries per week were most comfortable with wearing radiation protection equipment (p=0.000), wore it more often (p=0.000), and suffered more often from back pain compared to surgeons performing 1-7 surgeries or over 10 surgeries (p=0.000) (Figure 3).

It is also prudent to state that the above figures indicate a temporal relation between apron usage and back pain, which may point toward the fact that apron wearing is one of the factors that cause back pain in orthopedic surgeons. Another important finding was that the most common complaint of surgeons suffering from back pain was ill-fitting lead aprons rather than lead aprons being heavy (40% versus 34.2%) (p=0.043) (Table 2).

**Table 2:** Cross tabulation between back pain and problem

			Do you suffer from back pain or neck pain?				Total
				No	Sometimes	Yes	
Problem	Heavy	Count	0	85	75	83	243
		% within problem	0%	35%	30.9%	34.2%	100%
	Ill-fitting	Count	0	12	15	18	45
		% within problem	0%	26.7%	33.3%	40%	100%
	Decreased motion	Count	2	24	14	18	58
		% within problem	3.4%	41.4%	24.1%	31%	100%
	Total	Count	2	121	104	119	346
		% within problem	0.6%	35%	30.1%	34.4%	100%

Lead-free aprons (40%) and lead vinyl-based aprons (46.7%) were found to be more comfortable than lead-based aprons (24%) (p<0.01) (Table 3).

**Table 3:** Cross tabulation between types of apron design and feeling of comfortability

			Comfortability				Total
			Comfortable	Slightly uncomfortable	Very uncomfortable	Other responses	
What type of radiation protection apron do you use?	No response	Count	0	0	0	1	1
		% within What type of radiation protection apron do you use?	0%	0%	0%	100%	100%
	Do not know	Count	10	43	7	3	63
		% within What type of radiation protection apron do you use?	15.9%	68.3%	11.1%	4.8%	100%
	Lead based	Count	76	187	54	0	317
		% within What type of radiation protection apron do you use?	24%	59%	17%	0%	100%
	Lead-free	Count	8	10	2	0	20
		% within What type of radiation protection apron do you use?	40%	50%	10%	0%	100%
	Lead vinyl based	Count	7	7	1	0	15
		% within What type of radiation protection apron do you use?	46.7%	46.7%	6.7%	0%	100%
Total		Count	101	247	64	4	416
		% within What type of radiation protection apron do you use?	24.3%	59.4%	15.4%	1%	100%

**Figure 4:**
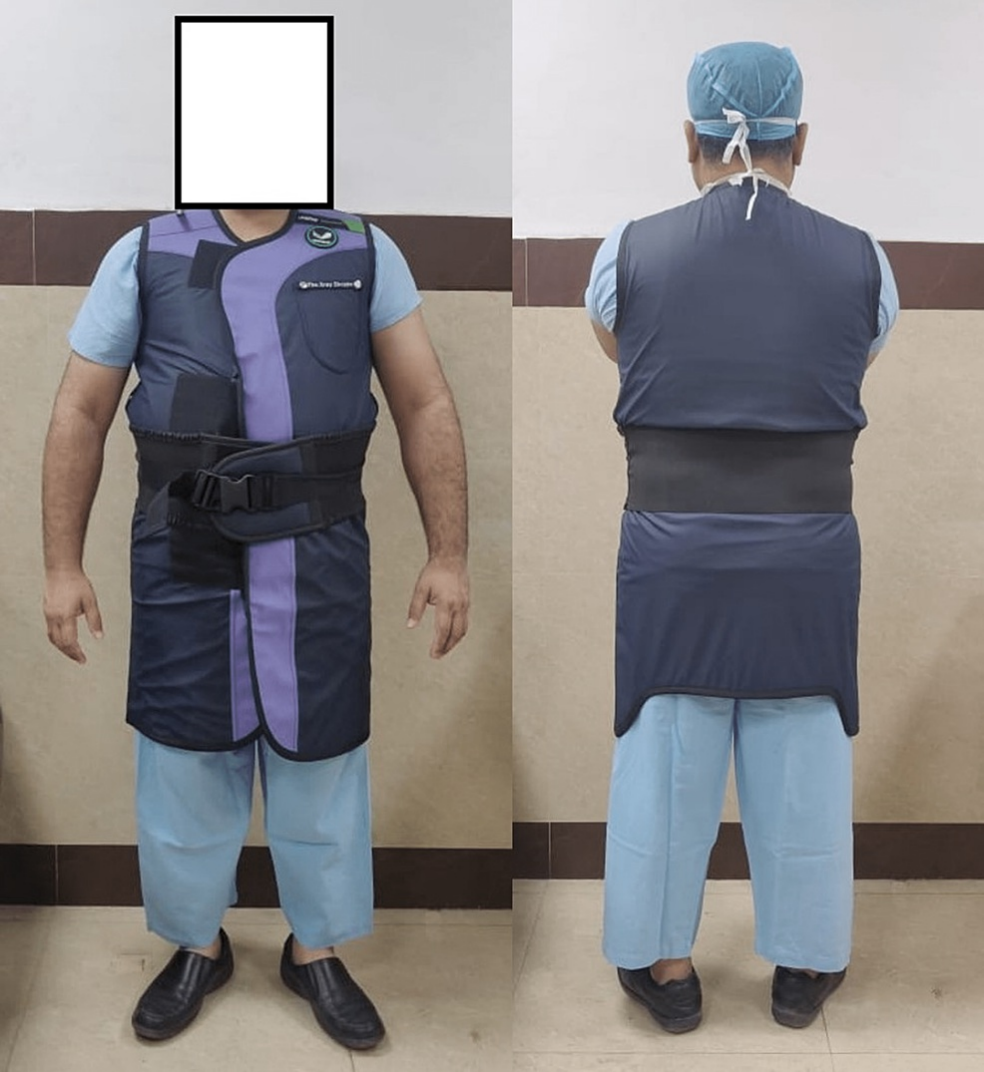
Single-sided coat type (front and back)

**Figure 5:**
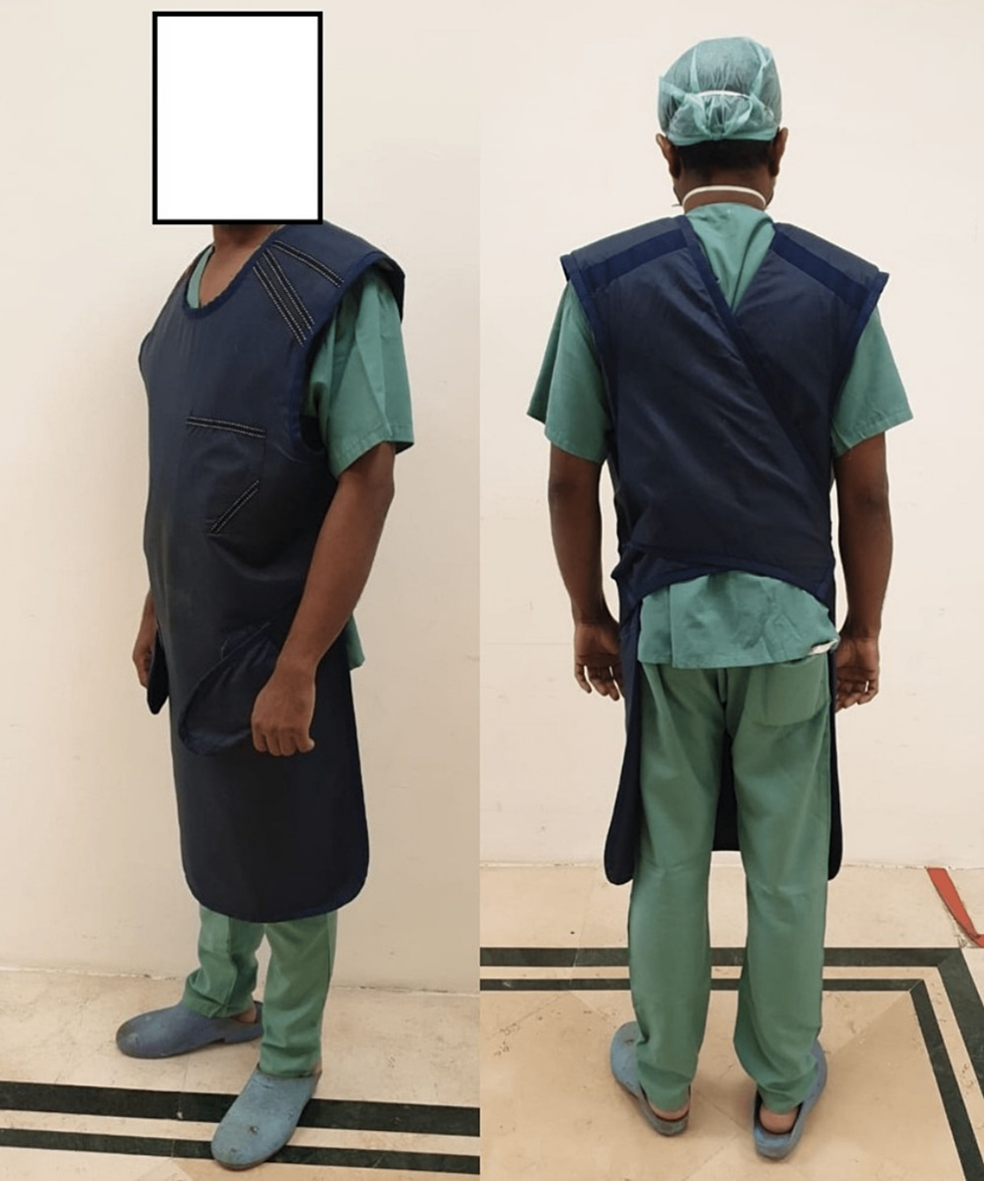
Wrap-around type (front and back)

Of our respondents, 63 (15.1%) did not know the type of apron they were using (Table 4 and Figures 4, 5). In our survey, the single-sided coat type design had more frequency of back pain compared to the wrap-around type (p=0.05).

**Table 4:** Types of apron compared with back pain

Type of apron		Number of responses that the majority complained of back pain (%)	p-value
Single-sided	289	109 (37.7%)	0.05
Wrap-around	100	31 (31%)	

We noticed that nine surgeons never wear radiation protection aprons and shields. Over 90% of these were from private practice (p<0.01), did less than two surgeries (p<0.01), and were aged >51 years (p<0.01).

## Discussion

Our survey shows that a very small proportion of orthopedic surgeons are comfortable with the current designs of radiation protection equipment. Furthermore, it reveals that very few surgeons are actually using thyroid shields. The survey is unique in telling the problems of such a large number of surgeons from multiple countries, which can be used as the basis for newer surgeon-friendly designs by radiation protection equipment manufacturing companies. Another important finding of our survey is that it describes two important factors that may be modified by the hospital administration and can help increase the usability of radiation protection equipment, i.e., temperature control and inability to call to mind to wear a thyroid shield. Of our respondents, 78% feel sweaty, and proper maintenance of operation theater (OT) temperature and humidity would make them more comfortable in wearing radiation protection equipment. Also, the majority of surgeons forget to wear thyroid shields, for which hospital administration should take proper steps including pasting of notifications at scrubbing counters and reminding surgeons to wear thyroid shields. Furthermore, manufacturers may incorporate thyroid shields in the apron, which may eliminate the chances of forgetting.

Concern with the fitting of aprons being more significantly linked to back pain than heaviness is a point worth mentioning. This factor is easily controllable, and back pain incidence may decrease substantially if hospital administration can provide better fitting aprons and manufacturers may increase modularity in designs. Lastly, our survey identifies high-risk groups of surgeons, those in private practice, doing less than two surgeries, and were more than 51 years old, that are not wearing radiation protection equipment; proper programs and workshops may be implemented by hospital administration targeting these groups and to sensitize them to use radiation protection equipment.

Currently, radiation safety has been of significant importance as the number of minimally invasive procedures being performed in orthopedic operation theaters has been increasing [1,2]. Surgeons are mainly affected by the scattered radiation. Aprons and thyroid shields have been designed to prevent the ill effects of this radiation [1]. However, they have their own problems. The earliest study of musculoskeletal issues of interventionists was reported in 1992 when Moore et al. [6] surveyed 236 radiologists regarding back pain and the use of lead aprons. Ross et al. [2] noted that lead apron-wearing cardiologists “reported more neck and back pain, more subsequent time lost from work, and a higher incidence of cervical disc herniations, as well as multiple-level disc disease.” The article noted that “interventionalist’s disc disease” is a confirmed entity and that it is possibly a consequence of lead apron use. Since then, numerous reports have identified them to be responsible for back and shoulder pain. However, most of these reports are for radiologists, cardiologists, and spine surgeons, and none for orthopedic surgeons, who are believed to wear radiation protection equipment only for shorter spells [2,6-9]. Ross et al. [2] compared back pain among radiologists and orthopedic surgeons and reported that 82% of orthopedic surgeons wear lead aprons with an average of 2.9 orthopedic surgeries per week. Contrary to these findings, our survey shows that 100% of surgeons use C-arm, 91% wear lead aprons, and 74.4% do more than three surgeries per week. It shows that the time and frequency of apron use has increased among orthopedic surgeons, and they are now more prone to occupational back pain.

Our survey showed that 63% of the respondents suffer from back pain or neck pain, which is similar to rates reported for interventional radiologists [7,8]. Livingstone et al. [7], in a survey of 91 interventional radiologists, reported that 47% had some sort of back pain, while Morrison et al. [8] reported that out of 640 interventional radiologists who were surveyed, 61% had back and 59% had neck pain. Our data indicates almost the same incidence of back pain among orthopedic surgeons as seen in interventional radiologists.

Contrary to Morrison et al. [8] who did not find age to influence back pain in interventional radiologists, we found that the 31-40 years age group is most prone to develop back pain. This may be because Morrison et al. [8] have used only two age groups (less than 50 and more than 50), and we used six different subgroups. Furthermore, this was the age group in our study that most often used lead aprons. Similar to the findings of Ross et al. [2] and Livingstone et al. [7], we also observed that back pain was proportionate to the usage of lead aprons. However, there are other studies in the literature that have shown no difference between the prevalence of back pain and the duration of lead apron use [5,6].

We also found that lead-free and lead vinyl designs were more comfortable compared to lead-based designs. Similar findings were reported by Morrison et al. [8]. Contrary to the report by Livingstone et al. [7], who reported that 29% of radiation interventionists were unaware of the type of radiation apron they were using, we found that 15% of the surgeons in our study were unaware of the type of apron they were using. It has been published in the literature that although the users claim to store the lead aprons correctly after use, this has not been the case [5]. Correct storage of lead aprons has been reported to be crucial in preventing cracks or holes from developing, which can reduce their integrity and hinder their protective ability [5,10]. The radiation safety officer of the hospital must impart knowledge to orthopedic surgeons in this aspect to further improve these numbers. Education improved the knowledge and understanding of lead apron use [5].

Livingstone et al. [7] reported that about 47% of their respondents have some kind of body aches due to wearing single-sided aprons. Similar to them, we also found single-sided aprons to be more often a cause of back pain compared to wrap-around type. We believe that this may be due to the lumbosacral support type belt provided in wrap-around aprons. A study showed adding a belt decreased spine load substantially and was associated with significantly less pain [11,12]. Other studies have not shown a significant difference in pain or disability between one- and two-piece garments (e.g., apron versus skirt and vest) [12,13].

Bowman et al. [14], in their nationwide survey of orthopedic residents, observed that forgetting was the number one reason to not wear a lead apron. Our survey showed that only 10.1% forget to wear lead aprons. This may be because most respondents in our survey were older, with more experience than residents, and therefore had a lower tendency to forget. However, surprisingly, over 35% of these respondents forget to wear thyroid shields.

Alexandre et al. [15] found that the use of lead aprons increases body temperature by 0.55°-0.95°. Similar to their findings, we also found that 78.8% of the respondents felt sweaty while wearing lead aprons. These two points must be kept in mind by the hospital administration. By providing proper operation theater (OT) temperature and humidity and pasting notification in scrub areas about wearing thyroid shields, we believe compliance to use radiation protection equipment may get better. Furthermore, surgeons in the government sector complained of sweatiness more often than those in the private or mixed sector, so it seems that OT temperature was more of a problem in the government sector, probably highlighting the need to maintain comfortable temperatures in the OT in government-owned hospitals.

Our study also observed that surgeons aged >51 years mainly involved in private practice and doing less than two surgeries per week are at the highest risk of not wearing radiation protection equipment, and therefore, measures to educate this subgroup regarding occupational radiation hazards may be beneficial in increasing compliance in using radiation protection aprons.

To the best of our knowledge, we did not find any survey of orthopedic surgeons explaining the problems they face while wearing radiation protection equipment. This survey showed that the heaviness of lead aprons and decreased movement during surgery were major causes of uncomfortability among surgeons. Similarly, thyroid shields were found suffocating by most surgeons, while in others, it irritated the skin or beard. These points may be kept in mind by manufacturing companies while designing radiation protection equipment.

Being the largest survey of orthopedic surgeons described in the literature that has assessed the usage of radiation protection aprons and shields is one of the important strengths of the study. Our study had various limitations as well. Firstly, it being a cross-sectional survey, the level of evidence is low. Secondly, the survey opinion is predominated by males and may not be representative of all orthopedic surgeons. Thirdly, a number of problems are inherent in any epidemiological study of back pain as the symptom is subjective and multifactorial. Therefore, all confounding factors can never be accounted for, and therefore, establishing a causal relationship may be difficult. Lastly, there are also problems associated with studying the use of lead aprons. Not all lead aprons are alike. Variations in weight and structure could significantly alter stresses on the back. Another limitation of our study is the lack of validation of our questionnaire. However, it was devised through extensive research by the author group and was based on a previously published questionnaire by Moore et al. [6]. Despite these shortcomings, this survey is one of the first attempts to highlight the problems orthopedic surgeons face while using radiation protection equipment.

## Conclusions

The study assessed the practice of orthopedic surgeons using radiation‑protective lead aprons and thyroid shields with the help of a simple questionnaire. The majority of the surgeons responded that they were not comfortable with the current designs. Thyroid shields are worn less than aprons by orthopedic surgeons. Lead apron weight and thyroid shield design ergonomicity were the number one reason for not wearing the radiation protection equipment. Among those who regularly wore aprons, a large proportion suffered from back or neck pain.
